# A New Model for a Carpool Matching Service

**DOI:** 10.1371/journal.pone.0129257

**Published:** 2015-06-30

**Authors:** Jizhe Xia, Kevin M. Curtin, Weihong Li, Yonglong Zhao

**Affiliations:** 1 Department of Geography and GeoInformation Science, George Mason University, Fairfax, Virginia, United States of America; 2 School of Geography, South China Normal University Tianhe Park, Tianhe, Guangzhou, China; Beihang University, CHINA

## Abstract

Carpooling is an effective means of reducing traffic. A carpool team shares a vehicle for their commute, which reduces the number of vehicles on the road during rush hour periods. Carpooling is officially sanctioned by most governments, and is supported by the construction of high-occupancy vehicle lanes. A number of carpooling services have been designed in order to match commuters into carpool teams, but it known that the determination of optimal carpool teams is a combinatorially complex problem, and therefore technological solutions are difficult to achieve. In this paper, a model for carpool matching services is proposed, and both optimal and heuristic approaches are tested to find solutions for that model. The results show that different solution approaches are preferred over different ranges of problem instances. Most importantly, it is demonstrated that a new formulation and associated solution procedures can permit the determination of optimal carpool teams and routes. An instantiation of the model is presented (using the street network of Guangzhou city, China) to demonstrate how carpool teams can be determined.

## Introduction

The work commute trip has long been known to be a critical element of transportation planning, and the most prominent contributor to traffic congestion. Since most commuters travel at regular times of day, this commonly leads to the morning and evening rush hours. The resulting congestion presents a cost to commuters in and of itself, but also influences pollution levels, energy consumption, and other related externalities. According to a report from the World Resources Institute (WRI), transportation accounts for nearly 14% of total greenhouse gas (GHG) emissions [1]. In addition to these economic and environmental concerns, energy efficiency—particularly associated with vehicle use—has become a high priority for governments around the world.

Numerous studies have been conducted to address inefficiencies in the traffic system. Models and solution procedures have been proposed to better manage the transportation system, including research into signal control optimization [2–3], and the integration of knowledge-based decision support systems [4]. Carpooling is another effective means of traffic management, through the direct reduction of the number of vehicles participating in the transportation system. Carpooling is loosely defined as the cooperation of two or more persons regarding the use of a single vehicle to meet their mutual commuting needs. In addition to the societal benefits outlined above there are potential benefits to the individuals who participate in the carpool. These benefits could include reduced fuel costs, reduced toll costs, reduced time spent in the commute (if high-occupancy vehicle (HOV) lanes are available for use), and potentially reduced driving stress for the passengers in the vehicle.

Carpooling is officially sanctioned by most governments. Numerous HOV lanes have been constructed or designated for carpooling commuters. As of 2012, 345 HOV facilities (in operation or in some stage of construction or planning) have been identified across the metropolitan areas of the United States [5]. However, research has shown that carpooling has been decreasing precipitously [6]. Low fuel prices, high-quality transportation facilities (roads), strong incomes, and issues regarding quality of life are believed to have led to this decrease [7–8]. Recently, with increasing fuel prices, rising environmental awareness, and an increasing number of policies directed toward carpooling activities, the awareness of the potential benefits of carpooling is believed to be increasing. A number of carpooling services have been built to provide carpool matching, such as Carpool World, Carpool Zone, and local government services. It has been found that a number of preference and policy variables can influence the success of these programs [9]. However, most carpooling services emphasize text-based information matching and integration, and seldom address the network analytic components of carpooling. Text-based carpool matching may result in a bad combination of carpooling team members, with high commute route costs. A network based carpool matching mechanism is needed to help commuters construct a carpooling team with minimum commute route costs.

In this paper, network-based carpool matching issues are addressed. In the literature review section, it is demonstrated that 3+ person carpooling is more effective in traffic management compared to 2-person carpool teams, but models, algorithms and services for 3+ person carpooling are not generally available due to the combinatorial complexity of the problems. This limitation motivates the research presented here, where a network-based carpool matching model for 3+ person carpooling is provided. In addition to the model, a range of solution procedures (both optimal and heuristic) are tested to determine their value in solving problems of this type. This paper is organized as follows: in Section 2 a review of the relevant literature regarding carpooling and carpool matching services is provided. Section 3 outlines a model for carpool matching and the variant solutions methods are applied to the carpool matching problem. Section 4 presents the experimental results across a large range of carpool matching problem instances, and Section 5 presents a practical application of the problem with a case study in Guangzhou city, China. Section 6 provides conclusions regarding the potential significance of these methodologies for policy and practice going forward, and suggestions for future research.

## Literature Review

### 2.1 History and the current state of carpooling

Articles [6–7] provide extensive reviews of the history of carpooling activity in the United States. Since the world oil shortages in the mid-1970s, Federal and local governments have implemented a variety of policies and programs to encourage carpooling activity. After that period, carpooling became increasingly popular in United States, with nearly 20% of commute trips using a carpool in the 1970s. However, this decreased precipitously after 1980, and has dropped to approximately 10% in 2010. Decreasing oil prices, improving transportation facilities, and increasing incomes are potential reasons for this decrease in carpool trips.

To encourage carpooling, many transportation agencies have built extensive networks of HOV lanes. However, the efficiency of–and benefits from—HOV lanes remain topics of controversy [10]. [11] investigated the influence of carpool lanes on overall transportation network performance. That research found that, using actual trip data, the presence of a reserved carpool lane on a congested highway can increase commute time. Similarly, [12] have found that the overall efficiency of a highway decreases with the presence of an HOV lane. It has been observed that HOV lanes frequently carry fewer people than general-purpose lanes. [13] demonstrated that a high proportion of two-person family carpools, also known as “fampools”, dramatically reduced the expected benefit of HOV lanes that were built with the expectation of carpools or vanpools carrying three persons or more per vehicle. This finding provides a primary motivation for the research presented here. If efficient means of generating larger carpools can be implemented, the expected benefits of HOV lanes can be more easily realized. Carpool matching services can lead to larger carpool sizes, and the ability to efficiently match multiple carpool users effectively multiplies the benefits of the carpool. This research presents methods for doing so.

### 2.2 Factors influencing carpooling activities

In the process of designing a carpooling matching service, it is critical to understand the factors involved in carpooling activities; including people’s perceptions of the benefits and costs of carpooling, and their concerns regarding safety. [14] conducted two surveys to analyse people’s views regarding carpooling activities. 40 undergraduate students (20 male, 20 female) and 48 staff members from the University of Iowa were selected, and asked to rate their concerns regarding carpooling. Responses were collected through both questionnaires and telephone survey techniques. The author concluded that the cost of time and convenience are the two decisive factors in the decision to carpool. People also expressed concerns regarding personal comfort and the gender mix among the carpool participants. [15] conducted a survey based on a significantly larger sample. The survey was conducted from May to July 2006 through the internet, targeting commuters in Dallas-Fort Worth and Houston. In total 4,634 responses were collected. Among those, 69.2% were solo drivers, 13.3% participated in a two-person carpool, 4.4% participated in a three-person carpool, and 2.3% rode in a vanpool. Results showed that access to HOV lanes and a reduction in driving stress were the two most important reasons for carpooling. Among the respondents who did not carpool, the most important reasons for driving alone were 1) the difficulty in finding someone with the similar location and schedule (55%), 2) the flexibility of solo driving (45%) and 3) needing a vehicle during the day (39%). The survey also showed that people are familiar with each other in most carpooling teams (75%). A survey (a total of 996 respondents) conducted in Lisbon, Portugal showed that the poor carpooling schedule and trust level between strangers are two major obstructions for carpool activities [16].

Location and schedule requirements seriously limit the convenience and flexibility of carpools. Although a large proportion of commuters likely share a similar commute route and schedule (thus leading to rush hour traffic), it is difficult for them to find each other and coordinate their travel. Therefore, an efficient carpool matching service which enables commuters to develop a potential carpool team can be a critical element in encouraging carpool use.

### 2.3 Carpool matching models and services

Numerous carpooling services have been developed with a wide range of approaches and functions. In this review, these services are loosely grouped into four categories: 1) services that list information but provide no explicit matching, 2) hardware- and communications-focused approaches, 3) services that use spatial information but not network-based information, and 4) services that use network-based spatial information to match users and provide good carpool routes. Each of these groups of services is reviewed in turn.

#### 2.3.1 Carpool matching through lists of users

The first category of carpool matching services contains those that provide information regarding users who wish to carpool, but provide no explicit carpool matching services. In this category, a number of carpooling services are based on textual information posting, searching, and integration. For example, Craigslist (www.craigslist.org) features free online classified advertisements and provides carpooling information for users in different metropolitan areas.

The main difficulty of such carpool services is that the system rarely suggests a match between drivers and riders. Rather, it simply provides a listing of those who may want to carpool, and the users need to search on their own to find other users with similar commute patterns. No actual carpool matching takes place through the system. If end users do successfully find a carpool team (often after significant trial and error using personal knowledge and perceptions of locations) they have no way of quantifying how efficient their carpool team is, nor can they view alternative carpool team options. These factors may combine to result in inefficient combinations of carpool teams.

#### 2.3.2 Hardware- and communications-focused approaches

[17] designed an architecture to assist commuters in defining a group of people who usually have similar commute routes and schedules. This architecture is primarily hardware-based; using a carpool device to collect daily commuter routes and schedules. This idea can be extended for use with smartphones. PASS [18] is a parking-lot-based method for carpooling which differs from vehicular ad hoc networks. Wireless devices and accelerator sensors are used to collect commuters’ information, and then establish a routing tree to deliver vehicle trajectory information to nearby parking lots. [19] investigated the network communications and the feasibility of using WiFi for carpooling activities in metropolitan areas. [20] employed a vehicle-to-passenger communications (V2P) approach to support communications between riders and drivers. These hardware- and sensor-based carpool matching approaches provide promising methods for the collection of commute information and they encourage communications between potential carpoolers. However, explicit matching algorithms are not their focus and commuting routes for suggested carpools may not be optimized.

#### 2.3.3 Services that use spatial information but not network based matching

In contrast to those discussed above, examples of systems such as the Seattle Smart Traveller (SST) [21] system provide more structured carpool matching services, although not based on network information. SST not only collects spatial and temporal commute information, but also performs matching using SQL specifications. A temporal match is performed to define the overlap in departure and arrival times, followed by a spatial match that is based on the intersection of the buffers centred on commuter locations. Carpool World (www.carpoolworld.com) provides worldwide carpool matching functions including scheduling, consideration of special needs, travel behaviour recording and address matching. This matching algorithm is mainly based on the latitude and longitude of commuters rather than matching based on the commuter route itself. Zimride (http://www.zimride.com) is another schedule-based carpooling service. The system is focused on long-distance carpool matching based on the driver’s schedule and the distance between driver and passenger.

#### 2.3.4 Services that use network-based spatial information to match users and provide good carpool routes

WIGEOPOOL [22] is a GIS-based traveller information system that performs carpool matching based on network locations. It provides a number of carpool services such as driver and passenger searching, carpool team matching, and commute cost sharing. Carpool Zone (www.carpoolzone.smartcommute.ca) is a carpool matching service which serves commuters in the greater Toronto and Hamilton area, Canada. It includes functions such as intelligent route matching, interactive mapping, pinpoint geocoding, and security, privacy, and administrative functions for the contacting and matching process [9]. Carpool Zone helps commuters to find other commuters who share a similar commuter route with specific needs (e.g. schedule, gender, language). The system gives the percentage of similarity between two commute routes through network analysis. “Let’s Carpool” [23] is a similar carpool matching service for the Wellington region of New Zealand. It provides similar functions, but the similarity between two commute routes is not given to the end users. An evaluation study of “Let’s Carpool” showed that the percentage of 1300 registered commuters who carpooled as their main commute mode increased significantly (from 12% to 27%) after the service was introduced.

“BlueNet” [24–26] is a carpool service in Taiwan with a mobile client and cloud-based carpool matching module. The system adopted a genetic algorithm approach in order to provide network-based carpool matching. [27] conducted a carpool matching simulation study based on 2008 travel demand data. Both matching strategies are designed to optimize the total system-wide vehicle miles travelled and the carpool matching rate, rather than individual preferences. Therefore, the interests of a single carpool user may not be guaranteed from a personal perspective. Taking the success of the above carpool studies, a number of mobile-based carpool applications have been developed, such as Lyft (https://www.lyft.com), Flinc (https://flinc.org), Carma (https://carmacarpool.com), and Tripda (www.tripda.com). These mobile applications provide basic real-time and network-based matching services, and improve the user experience by introducing driver-passenger communication tools, payment modules (e.g., fee calculator and payroll system), and security background check functions.

These carpool services use network-based algorithms to suggest a carpool team and commute route. The matching algorithms are mainly designed for a single driver–and a single passenger or system-wide carpool matching [24–27] optimization. In other words, the algorithms and carpool matching service may not provide an optimized solution when a commuter wants to carpool with more than one person. [12–13] have argued that two-person carpooling is so inefficient that it may even be considered a burden to the transportation system. Three-person carpooling and vanpooling are recommended. Models, algorithms and services for 3 + person carpooling are needed, but these are still missing in the current carpooling research and literature. This limitation motivates the research presented here, where a network-based carpool matching model for three or more person carpooling is provided. Moreover, in two recent reviews of issues surrounding ridesharing [28] and methods for optimally addressing ridesharing [29] systematic classifications were made in order to “foster the development of effective formal ridesharing mechanisms” [28]. This work presents an effort to develop one such formal mechanism and evaluate its potential.

## Methodology

### 3.1 Conceptualization of Carpooling Activities on networks

Generally, there are two types of people who participate in carpooling activities: 1) “drivers” who have vehicles available for commuting and who want to share their vehicles with other commuters, and 2) “passengers” who are looking for a ride with drivers. Both the drivers and the passengers must participate in a two-sided matching in order to address all participants’ needs. Further, a carpooling activity can be abstracted into two fundamental processes: 1) a carpool matching process that enables drivers or passengers to develop a carpooling team, and 2) a daily commute process where the driver needs to choose the order in which they will pick up and drop off all team members.

After a carpooling team is formed, the driver must pick up all team members from their residences in a pre-determined order, then drop off all team members at their workplaces in a pre-determined order, ending at the workplace of the driver. This process must repeat in roughly the reverse order at the end of the work period. In this process, the “residence” or “workplace” could also be a group pick-up area (e.g. a commute pooling lot, an apartment building), or an inter-modal transfer location (e.g. a bus stop or a train station) if such locations facilitate the carpooling process.

Common constraints on the carpool matching and daily commute processes are as follows:

1. The total number of commuters in a carpooling team cannot exceed the maximum capacity of the commute vehicle.
2. The entire commute route must start at the residence of the driver and end at the workplace of the driver
3. Each team member must be picked up before they can be dropped off. While this constraint may seem obvious, it must be made explicit in the carpool matching model outlined below. However, this constraint does not require that all team members are picked up before some team members are dropped off. In other words, the driver could drop off a team member before picking up some other team member.

The objective of the carpool matching and daily commute processes is to minimize the cost (e.g. route length, commute time, or some combined cost) of the daily commute.

**Fig 1:**
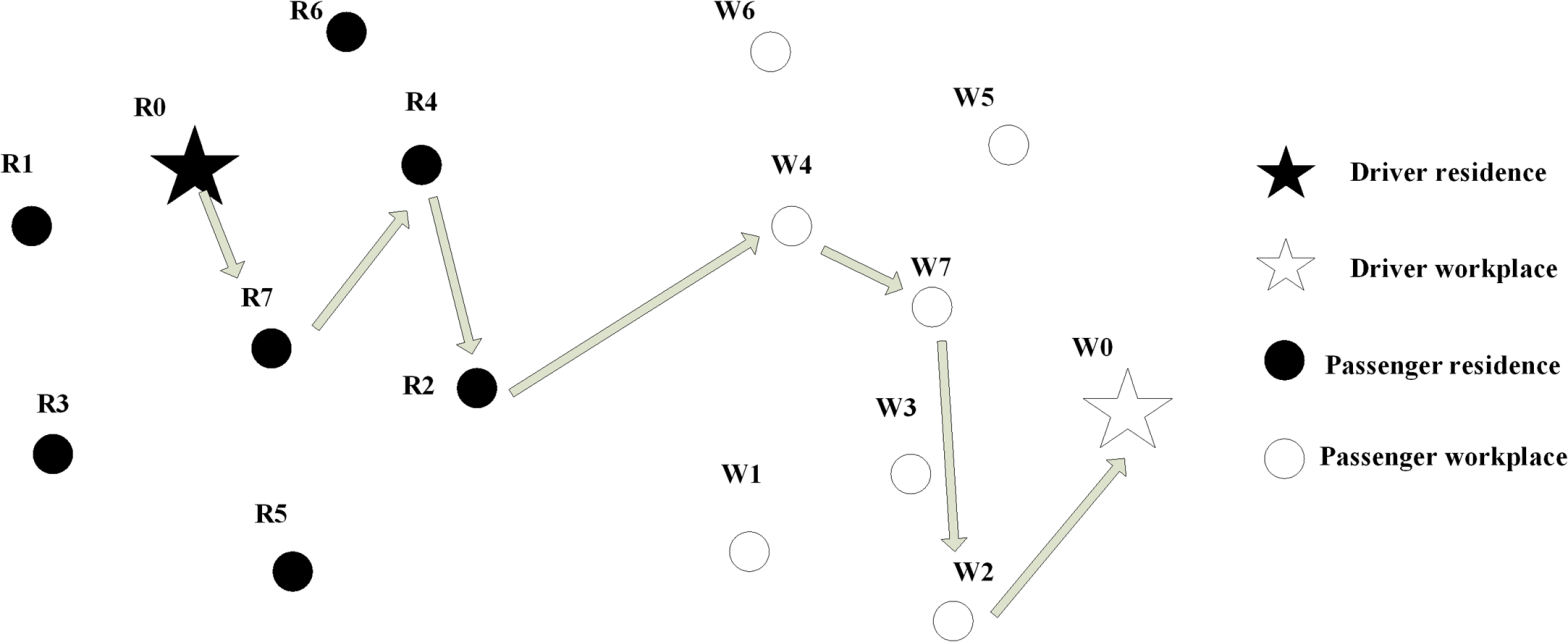
A sketch map for carpooling activity in a network.

Fig 1 shows an example of this carpooling activity model in a network. Each person in the example is identified by a number, and the residence and workplace of that person share the same number. For example, Person 4 is termed P4, and that person lives at location Residence 4 (R4) and works at Workplace 4 (W4). In this example, Person 0 (P0) is a driver and P0 initiated a carpool matching process. P0 chose P7, P4 and P2 to be his or her carpooling team members. The order for picking up those members is P0->P7-> P4->P2 and the order for dropping off is P4->P7->P2->P0. While this example shows only a single vehicle carpool matching process, an implementation of this system would permit many such carpools to be developed based on the needs of multiple drivers and many potential passengers.

### 3.2 Basic Assumptions

The network-based carpooling model has the following assumptions:

1. The carpooling model operates on a graph, or network. Residence and workplace locations can be abstracted as points along the network. Roads between residences and workplaces can be abstracted as arcs.
2. All residences and workplaces are connected by roads. Therefore, the model assumes that all points can be connected by arcs. The arcs are undirected, meaning that travel is possible in either direction.
3. The impedance associated with any arc in the carpooling model can represent any cost function that is appropriate for the commuters. This can include the length of the road, the drive time (including congestion factors), road conditions, weather conditions, tolls, or any combination of cost factors that are pertinent to the commute. These impedances can be implemented by weighting the arcs or nodes in the network appropriately.

### 3.3 Optimal Solution Procedures

#### 3.3.1 Optimal solution through enumeration

For small problems of this type it is well known that trying every possible arrangement of carpool routes is a solution method that is guaranteed to provide the optimal solution. Known as enumeration or brute-force search, algorithms of this type would test every possible carpooling team combination and carpooling route in order to find the best selection of team members and the associated best carpooling route. For this problem the enumeration process began with the identification of the pool of potential candidate passengers, and the number of passengers to be chosen from that pool. Based on those inputs the following steps were performed: 1) for each unique set of possible passengers out of the candidate pool identify all possible sequences of pick-ups from home locations and drop-offs at work locations; 2) eliminate any sequences where a passenger is dropped off prior to being picked up; 3) for the remaining logical sequences compute the shortest path; 4) choose the minimum shortest path; 5) return to step 1 and repeat the process for each unique set of passengers from the pool.

The scale of the problem follows the following equation given the number of carpool members (s1) and potential candidates (*K*):

$$Problem\;Scale=C_{k}^{s1}\;P_{s1*2}^{s1*2}$$ (Equation 1)

Assume there are 50 candidates and a driver wants to select three passengers for the carpooling team. There will be $C_{50}^{3}$ options for passenger selection, and $P_{6}^{6}$ permutations for picking up and dropping off those passengers. The magnitude of the set of possibilities will be $C_{50}^{3}\;P_{6}^{6}\;=\;14,112,000$, team/route combinations. If there are 100 candidates and 3 other carpool members, the magnitude of the set of possibilities will be $C_{100}^{3}\;P_{6}^{6}$ = 116,424,000 carpool/route combinations. If there are 50 candidates and 4 other carpool members, the magnitude of the set of possibilities will be $C_{50}^{4}\;P_{8}^{8}\;=\;9,285,696,000$ team/route combinations.

As can be seen–and as expected with an enumerative approach to solving these problems–the problem scale dramatically increases according to the increase in the number of candidates and the number of passengers in the carpool. In a real-world carpool matching process, there could be thousands of candidates. One would expect that any brute-force algorithm would require far too much computing time and resources for model execution on a problem instance of any significant size. Therefore a more efficient algorithm is needed for providing a real-world carpool matching service. Both a more efficient optimal solution procedure and heuristic approaches are outlined below.

#### 3.3.2 Linear Programming solution procedure

Integer linear programming provides a means of determining optimal solutions to problem instances that are beyond the reach of brute-force algorithms. In this section, a mathematical formulation of the carpooling route selection problem is presented and discussed.

Consider the following set of notation:

*p* is the index of passenger candidates and the driver; *p* = 1, 2, …, *N*, where *N* is the total number of passenger candidates plus the driver;

*k* is the index of residences; *k =* 1, 2, *…*, *N;*

*l* is the index of workplaces; *l = N+1*, *N+2*,*…*, N*2;

It is important to note that the indices of passengers, residences, and workplaces are all ordered. That is, passenger 1 (*p* = 1) lives at residence 1 (*k* = 1) and works at workplace *N* + 1 (*l* = *N* + 1).

Additionally, the residence of the driver is denoted as *s*, and the workplace of the driver is denoted as *t*, where *t* = *s* + *N*;

*i* and *j* are indices of nodes (residences or workplaces); *i*, *j* = 1, 2, …, N*2; following from above the node is a residence when *i*, *j* < = *N* and the node is a workplace when *i*, *j* > *N*;

*d*_*ij*_ is the cost of the arc (e.g. length, impedance, or some other user defined cost) between node *i* and node *j*;

*C* is the number of passengers in the carpool team (defined by the driver based on the capacity of the vehicle);

*r* and *f* are indices of arcs comprising a route; *r*, *f* = 1, 2, …, *R*;

*R* is the number of arcs in a carpool route, *R* = C*2 +1;

*x*_*ijr*_ is a decision variable equal to 1 if the arc from *i* to *j* is chosen for step *r* in the route, and equal to 0 otherwise;

Using this notation, the general carpooling route selection formulation can be given as: Minimize

$$Z\;=\sum_{i=1}^{N*2}\sum_{j=1}^{N*2}\sum_{r=1}^{R}d_{ij}\;{\text{x}}_{ijr}$$ (1)

Subject to:

$$\sum_{i=1}^{N*2}\sum_{k=1}^{N}\sum_{r=1}^{R}x_{ikr}=C$$ (2)

$$\sum_{i=1}^{N*2}\sum_{l=N+1}^{N*2}\sum_{r=1}^{R}x_{ilr}=C+1$$ (3)

$$\sum_{k=1}^{N}x_{sk1}=1$$ (4)

$$\sum_{l=N+1}^{N*2}x_{ltR}=1$$ (5)

$$\sum_{i=1}^{N*2}x{\;}_{ikr}-\sum_{i=1}^{N*2}\sum_{f=r+1}^{R}\;x_{i\left(k+N\right)f}=0\;,\;for\;k=1,\;2,\ldots \;,\;N;k\;\neq s;\;r=1,\;2,\ldots \;,\;R-1$$ (6)

$$\sum_{i=1}^{N*2}\sum_{r=1}^{R}x_{ijr}\leq 1\;for\;j=1,2,\ldots ,N*2$$ (7)

$$\sum_{j=1}^{N*2}\sum_{r=1}^{R}x_{ijr}\leq 1\;for\;i=1,2,\ldots \;,N\;*\;2$$ (8)

$$\sum_{i=1}^{N*2}x_{ijr}-\;\sum_{i=1}^{N*2}x_{ji\left(r+1\right)}=0\;for\;j=1,2,\ldots \;,N*2;r=1,2,\ldots \;,R-1$$ (9)

$$\sum_{i=1}^{N*2}\sum_{j=1}^{N*2}x_{ijr}=1\;for\;r=1,2,\ldots \;,R,$$ (10)

The objective function (1) seeks to minimize the cost of the entire carpool route. Constraint (2) requires the driver to pick up a given number (*C*) passengers at residences and (3) requires that the driver must drop off the given number (*C*) passengers and the driver at workplaces. Constraint (4) requires that the carpool route must start with the drivers’ residence, and (5) requires that the carpool route must end with the drivers’ workplace. Constraint (6) ensures that the driver will not drop off a passenger before picking her up. In order to ensure a complete, connected, and non-overlapping carpool route, constraint (7) requires that no more than one arc entering any node be selected, constraint (8) requires that no more than one arc exiting a node be selected; and constraint (9) ensures that if an arc enters a node on step *r* of the route, an arc exiting that node must be chosen for step *r* + 1 of the route. Constraint (10) ensures that there will be exactly one arc chosen for any step in the route.

This formulation can be used to generate optimal solutions for problem instances of hundreds of candidates for the carpool teams. However, even this method fails to find solutions for larger problems due to the lack of computing resources, or the time needed to determine optimality. Therefore, a practical application of carpool routing will almost certainly benefit from the implementation of heuristic methods.

### 3.4 Heuristic Solution Procedures

It is well known that the demands on computer resources and solution times grow rapidly when searching for the optimal solutions to highly combinatorially complex problems. The search for suitable heuristic algorithms to resolve this issue has been ongoing for decades. While this is not the appropriate forum for a thorough review of heuristic techniques, those such as Tabu Search [30–31], ant colony optimization [32], genetic algorithms [33], and simulated annealing [34], are among the most prominent. For the carpool selection and routing problems addressed here, both a simulated annealing and a Tabu search heuristic have been implemented. In so doing, this research tests one method from the class of smart interchange heuristics (Tabu), and one method from the class of heuristics that are based on correlates to natural processes (simulated annealing), in order to explore the costs and benefits of each class.

#### 3.4.1 A Simulated annealing heuristic solution procedure

Simulated annealing heuristics simulate the physical process of annealing. When metal is heated to a very high temperature, its molecules can move freely. When the temperature falls, the whole system returns to thermodynamic equilibrium and the molecules will stay in their altered state. By simulating this physical process, a simulated annealing heuristic can effectively avoid premature convergence to a local optimum and therefore possibly find a solution closer to (or ideally equal to) the optimal solution.

**Fig 2:**
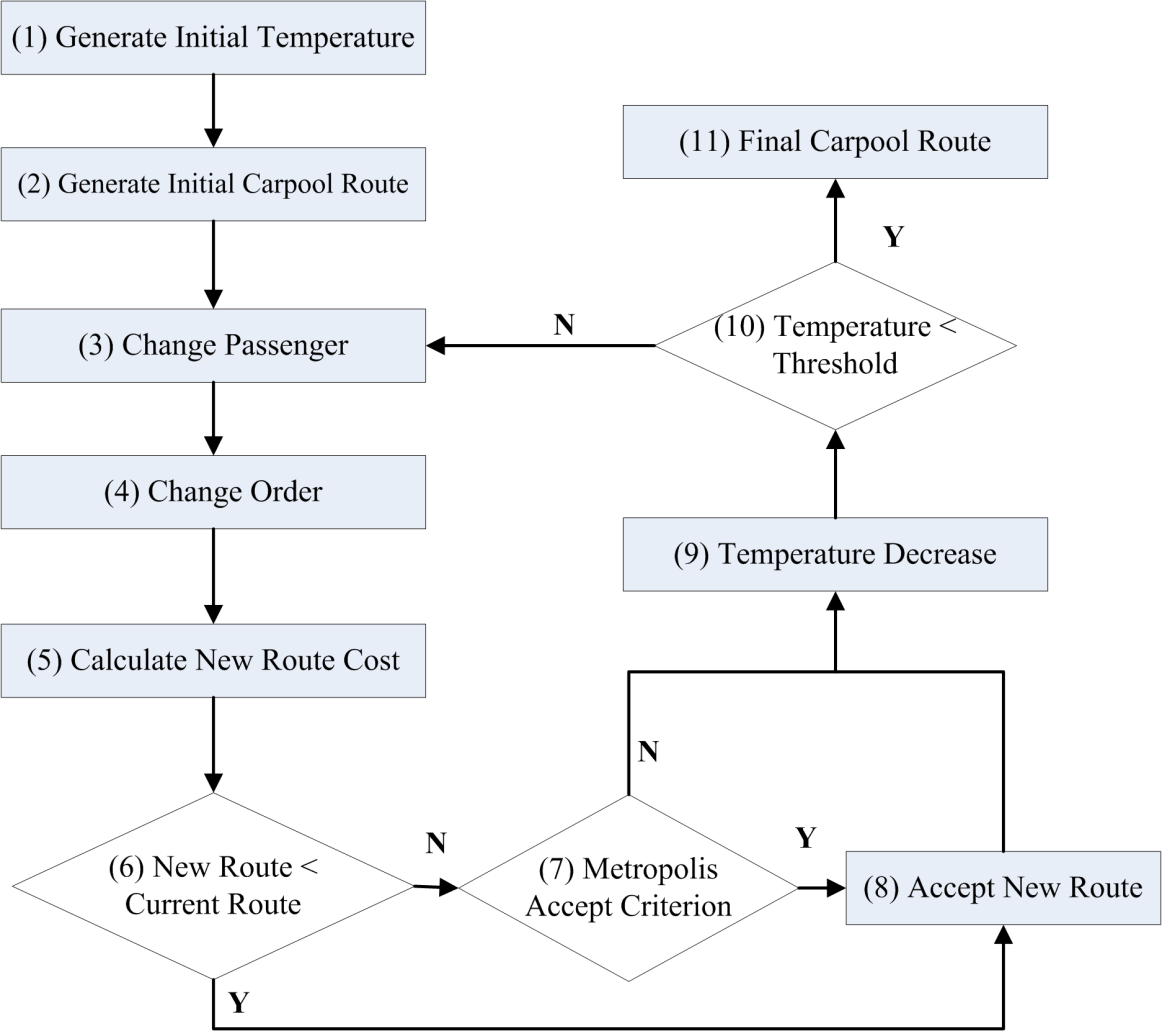
Workflow of simulated annealing carpooling route selection heuristic.

In the problem at hand, the length of the carpooling route corresponds to the energy of the metal, and the best route selection corresponds to the lowest energy state. We simulate the annealing process by setting the rule of temperature decrease and the rule for changing the carpooling route. Fig 2 shows the work flow of the simulated annealing carpooling route selection heuristic. The steps include: (1 and 2) generate initial temperature and initial carpooling route; (3 and 4) generate a new carpooling route by changing passengers and routes; (5) calculate the cost for the new route; (6, 7, and 8) if the new carpooling route is better than the current route, the current route will be replaced by the new route; otherwise, the new route will not be accepted if it also fails the Metropolis criterion test (Metropolis et al., 1953); (9) decrease the temperature; (10 and 11) when the temperature approaches a predetermined lower threshold, the current route will be chosen as the solution; otherwise, go back to step (3) to generate a new route. The pseudo-code of the simulated annealing carpooling route selection heuristic is shown in algorithm 1 to algorithm 4. The algorithm 2 is used for the Metropolis test in the simulated annealing process. The algorithm 3 and algorithm 4 are designed to determine all possible permutations of feasible carpooling routes given the current passenger pool. This function adopts a classic recursive permutation algorithm.

Algorithm 1. Carpool Matching Simulated Annealing

Input: Initial Temperature, CoolingRate, Threshold Temperture, Passenger Number (c), Candidate List (P1, P2, …, Pn), Distance Matrix

Output: Optimized Carpool Route

SA1: Passenger_Pool = Randomly c number of candidate

SA2: Current_Route = a random route to pick and drop off passengers

SA3: Best_Route = Current_Route

SA4: Current_Temperature = Initial Temperature

SA5: While (Current_Temperature > Threshold Temperture)

SA6: Passenger_Pool = Randomly replace a passenger with a candidate

SA7: New_Route = FindBestRoute (Passenger_Pool)

SA8: If (Cost (New_Route) < Cost (Current_Route))

SA9: Current_Route = New_Route

SA10: Else

SA11: Difference = Cost (Current_Route)—Cost (New_Route)

SA12: Metropolis = MetropolisCriterion (Difference, Current_Temperature)

SA13: If (Metropolis is True)

SA14: Current_Route = New_Route

SA15: If (Cost (Current_Route) < Cost (Best_Route))

SA16: Best_Route = Current_Route

SA17: Current_Temperature = Current_Temperature * CoolingRate

SA18: Return Best_Route

Algorithm 2. MetropolisCriterion

Input: Difference, Current_Temperature

Output: True/False

MC1: Metropolis = Math. Exp (Difference / Current_Temperature)

MC2: If (Metropolis > Math.Random())

MC3: Return True

MC4: Else

MC5: Return False

Algorithm 3. FindBestRoute

Input: Passenger_Pool

Output: BestRoute with Current Passenger_Pool

FR1: Initialize route list with passenger _pool // e.g, {Pick1, Pick2, Pick3, Drop1, Drop2, Drop3} for the order of picking up and droping off passenger 1, 2, 3

FR2: BestRoute = Permutate routers (route list, 0)

FR3: Return BestRoute

Algorithm 4. Permutate routers

Input: int[] route list, int start,

Output: BestRoute

PR1: If (start > = route list.length)

PR2: If (route list is feasible) // not drop off before pick up the passenger

PR3: currentDistance = Cost (route list)

PR4: If (currentDistance < BestRoute)

PR5: BestRoute = currentDistance

PR6: For (j = start; j < = route list.length—1; j++)

PR7: Swap the Step start and Step j in the route list

PR8: Permutate routers (route list, start+1)

PR9: Swap the Step start and Step j in the route list

PR10: Return BestRoute

#### 3.4.2 Tabu search solution procedure

Tabu search is a widely-used heuristic procedure that is designed to avoid local optimal solutions, and permit the search to find better–and perhaps global optimal–solutions. While we do not provide a comprehensive review of the method of Tabu search, fundamentally, a tabu list is utilized to record the recent history of the search and direct the search away from recent solutions in order to more broadly explore the solution space. Parameters are set that prevent cycling back to previously visited solutions for a period of time (number of iterations).

**Fig 3:**
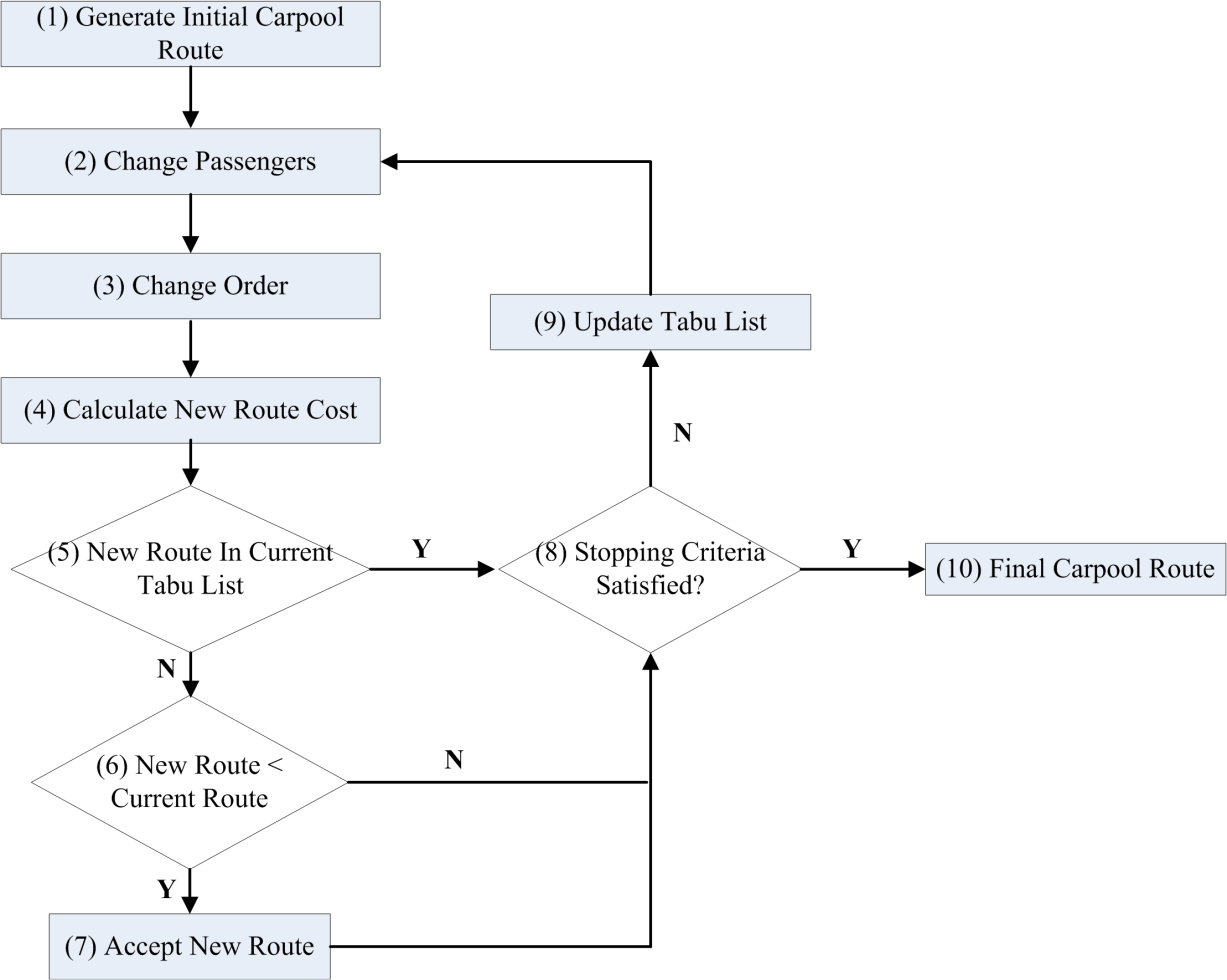
Workflow of Tabu search carpooling route selection heuristic.

Fig 3 shows the work flow of the Tabu search heuristic for the carpooling route selection problem. The steps include: (1) generate an initial carpooling route by randomly selecting passengers and routes; (2 and 3) generate neighbouring solutions (carpooling routes) by changing passengers and orders of picking up and dropping off; (4) calculate the cost for the new route; (5, 6, and 7) if the new carpooling route is not in the Tabu list and is better than the current route, the current route will be replaced by the new route; otherwise, go to step (8); (8 and 9) if the stopping criteria is not satisfied, update the Tabu list and go back to step (2) to generate neighbouring solutions; (10) Output the final carpool route once the stopping criteria is satisfied. The pseudo-code of the Tabu search carpooling route selection heuristic is shown in algorithm 5 and algorithm 6.

Algorithm 5. Carpool Matching-Tabu Search

Input: Tabulist_MaxSize, Passenger Number (c), Candidate List (P1, P2, …, Pn), Distance Matrix

Output: Optimized Carpool Route

TS1: Passenger_Pool = Randomly c number of candidate

TS2: Current_Route = a random route to pick and drop off passengers

TS3: Best_Route = Current_Route

TS4: Tabulist = null

TS5: While (! Stopping Criteria)

TS6: Local_Route = null

TS7: While (Neighborhood Searching Criteria)

TS8: Passenger_Pool = Randomly replace a passenger with a candidate

TS9: Current_Route = FindBestRoute (Passenger_Pool) // Algorithm3

TS10: If (Current_Route not in the Tabulist)

TS11: Local_Route = Current_Route

TS12: If (Cost (Local_Route) < Cost (Best_Route))

TS13: Add Local_Route to the Tabulist

TS14: Best_Route = Local_Route

TS15: While (Tabulist_Size > Tabulist_MaxSize)

TS16: Expire_Tabulist (Tabulist)

TS17: Return Best_Route

Algorithm 6. Expire_Tabulist

Input: Tabulist

Output: New Tabulist

ET1: Index = 0;

ET2: While (Index < Tabulist_Size)

ET3: Tabulist (Index) = Tabulist (Index + 1)

ET4: Index = Index + 1

ET5: Return Tabulist

## Implementation and experimental results

### 4.1 Experiment data and environment

The experiments were performed using both real-world and simulated data. For an underlying network representation the road network for Guangzhou city, China (S4 File) was employed. To determine the locations of carpool drivers and potential passengers, many sets of point locations were randomly generated within the bounding box of Guangzhou city. The sets contained an increasing number of potential passengers ranging from 50 to 1,000, with each set incremented by 50 (50, 100, 150, …, 900, 950, 1,000) (S1–S4 File).

The experiments were performed on a desktop machine, running Intel Quad Cores i7-3770 @ 3.4 GHz, with 12 GB RAM, a hard disk with 7200 rpm storage seeking speed, and the Windows 7 operation system.

### 4.2 Optimal solution results

**Fig 4:**
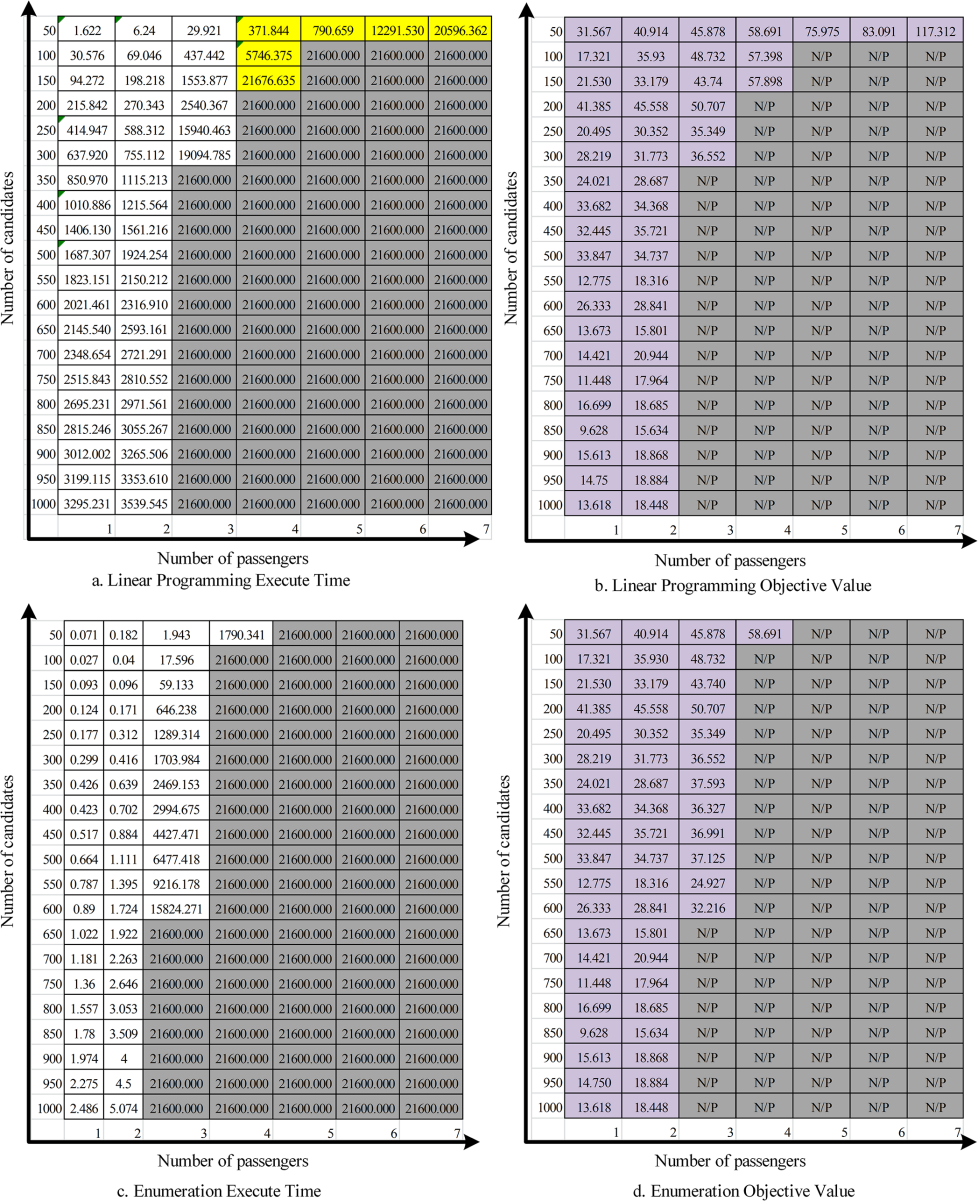
Optimal solution results.

Fig 4 shows the execution times and objective function values for the linear programming procedure and the brute force enumeration procedure.

In Fig 4, the yellow highlighted cells represent problem instances where the linear programming procedure either solved the instance optimally where enumeration could not (within a 6 hour window) or the optimal solution was determined faster through linear programming. The results are as follows:

- With small-scale carpool route selection instances (smaller than four passengers), the enumeration procedure can provide the optimal solution with a lower execution time as compared to the linear programming procedure. With three passengers, the linear programming procedure can only provide optimal solutions within six hours when the number of commuters is less than 300. The enumeration procedure can solve 3-passenger instances up to 600 commuters.
- As the number of passengers increases (above 3), the linear programming procedure can provide additional optimal solutions that are beyond the reach of the enumeration procedure, when a six-hour time limit is imposed.
- For the particular instance of 4 passengers and 50 potential commuters, both methods find the optimal solution within six hours, although the linear programming procedure does so significantly faster.
- The solution time for the linear programming procedure increases faster with the increase in the number of potential commuters, while the solution time for the enumeration procedure increases faster with the increase in the number of passengers in the carpool team.

### 4.3 Heuristic Solution and results

**Fig 5:**
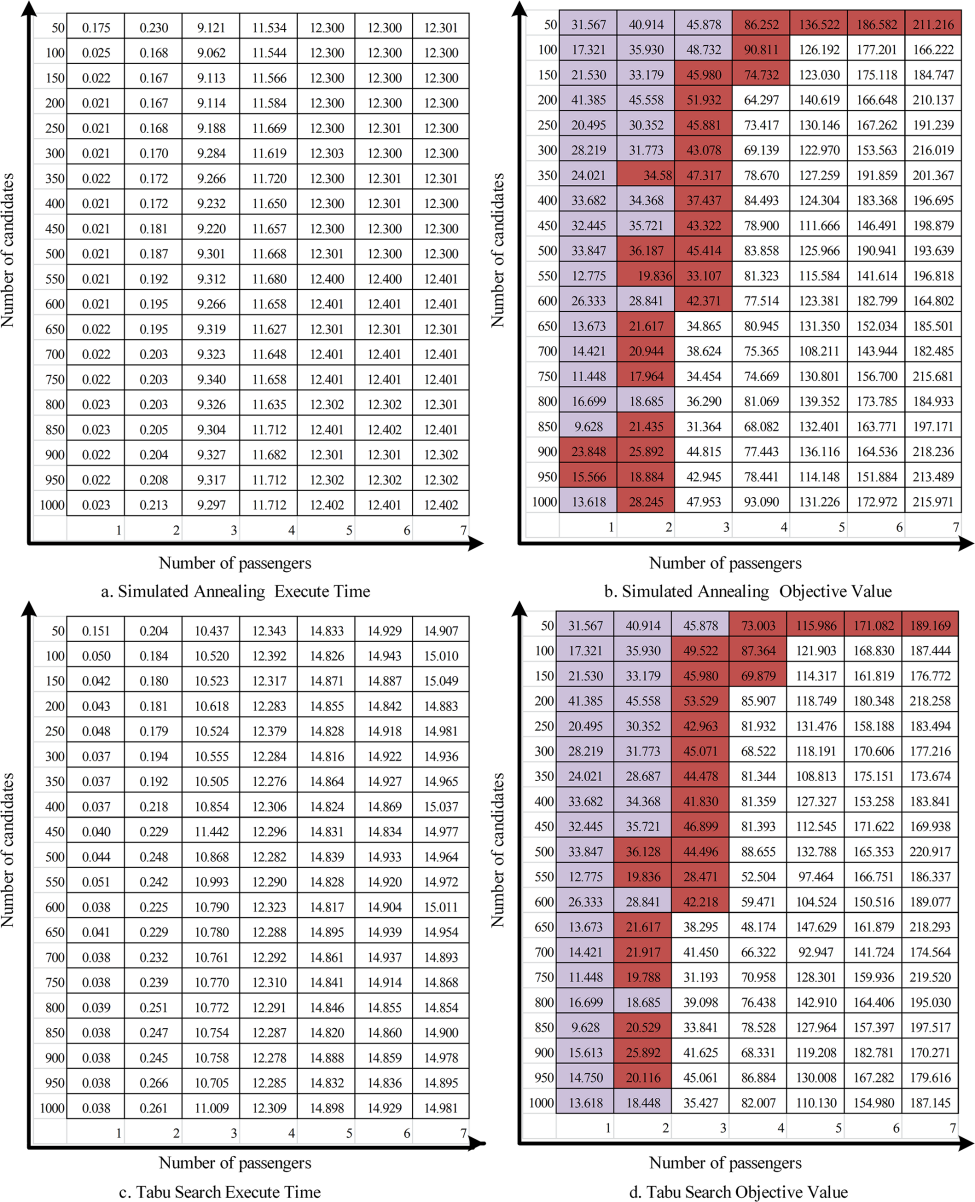
Heuristic Solution Results.

Fig 5 shows the execution times and objective function values for the simulated annealing and Tabu search heuristics.

The purple highlighted cells represent those instances where the respective heuristics are known to have identified the optimal solution. The red highlighted cells are those that are known to have identified sub-optimal solutions. The remaining values are the best solution found by the heuristic, although it is not known if the solution is optimal or not.

- In general, the Tabu search procedure requires more execution time than simulated annealing (but not by more than 3 seconds for any instance).
- When the number of passengers is smaller than 4, there is no significant difference between the Tabu search and simulated annealing results. However, Tabu search does find the optimal solution more often than simulated annealing (23 times v. 20 times out of 28 known optimal solutions).
- With larger problem size (number of passengers greater than 3), the Tabu search procedure generally provides a shorter carpooling route cost (about 5%), when compared with the simulated annealing procedure with similar execution time.

### 4.4 Comparison & Discussion

The results above have potentially significant consequences for the instantiation of a real-world carpool matching system. The consequences depend on the constraints under which the carpool matching must operate. For example, assume that a carpool is being established for long-term commuting purposes. A van holding 7 or more persons is to be employed for the commute, and there are 50 potential commuters. In this case, it appears that an optimal solution (and particularly the linear programming approach) is appropriate. The solution time limit of 6 hours would not be prohibitive (and could be increased, in fact), and the optimal solution would, of course, be guaranteed. The use of a heuristic solution procedure in this same case could result in a solution that is at least 61% above optimal (Tabu search returns 189 km while linear programming returns 117).

Conversely, if the carpools are to be determined on a daily/nightly basis, and only one or two passengers will be chosen, then the brute force approach is preferred. Finally, in the instances where larger carpools are desired, and the solution time is a concern, the heuristic approaches are the only option. Their rapid solution time allows for the near immediate determination of the carpool personnel and route, allowing for planning for the next day’s commute.

## The Carpool Matching System

While the results above demonstrate the relative value of different approaches to solving the carpool matching problem, we are also concerned here with the practical implementation of those approaches. In this section the architecture of such a system is outlined, with an example case study from Guangzhou city, China.

**Fig 6:**
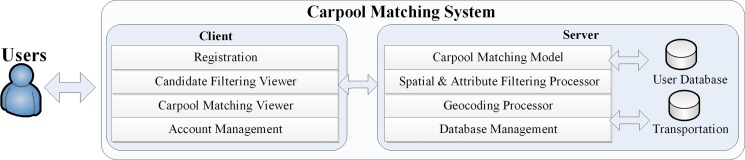
Architecture of a carpool matching system.

The architecture of a carpool matching system is shown in Fig 6. The carpooling matching service contains two tiers (a client tier and a server tier). The **Client tier** provides a Graphical User Interface (GUI) and all interactive functions that are necessary to initiate a carpool matching activity, including registration, account management, candidate filtering viewer, and carpool matching viewer. The **Server tier** includes the carpool matching model (as discussed above), the model processor, a filtering processor, and a geocoding processor to support the carpool matching function.

### 5.1 Implementation

**Fig 7:**
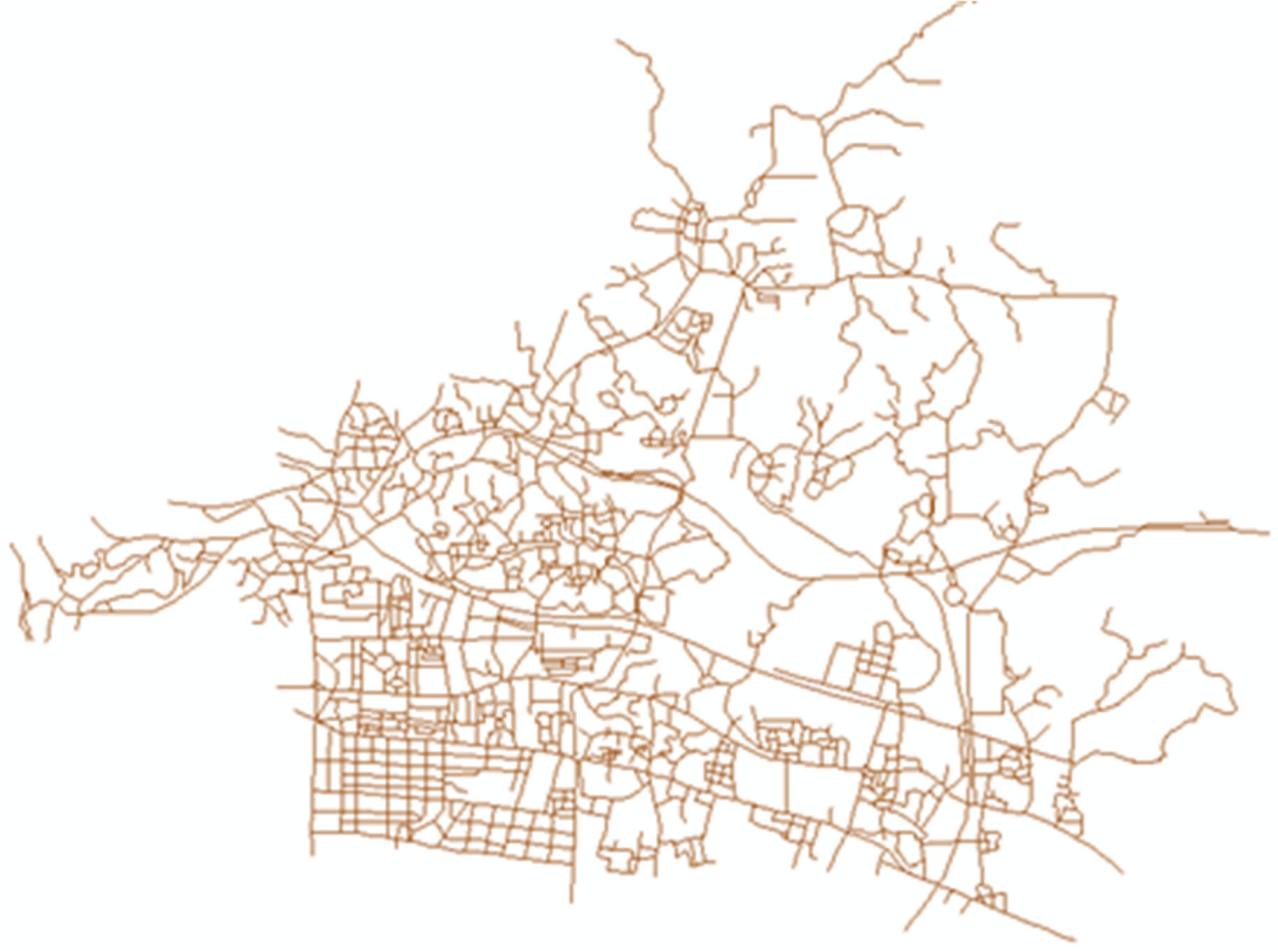
Road network of Guangzhou city in 2009.

A carpool matching system has been developed using the Microsoft.Net Framework 4. Microsoft SQL Server is used for user information storage and management. The ArcGIS Engine is used for spatial data management and visualization. The city road network for Guangzhou city is shown in Fig 7.

### 5.2 Use case

In this section, we describe a use case illustrating how the carpool matching system can support carpooling activity among commuters. The process includes:

#### Registration

**Fig 8:**
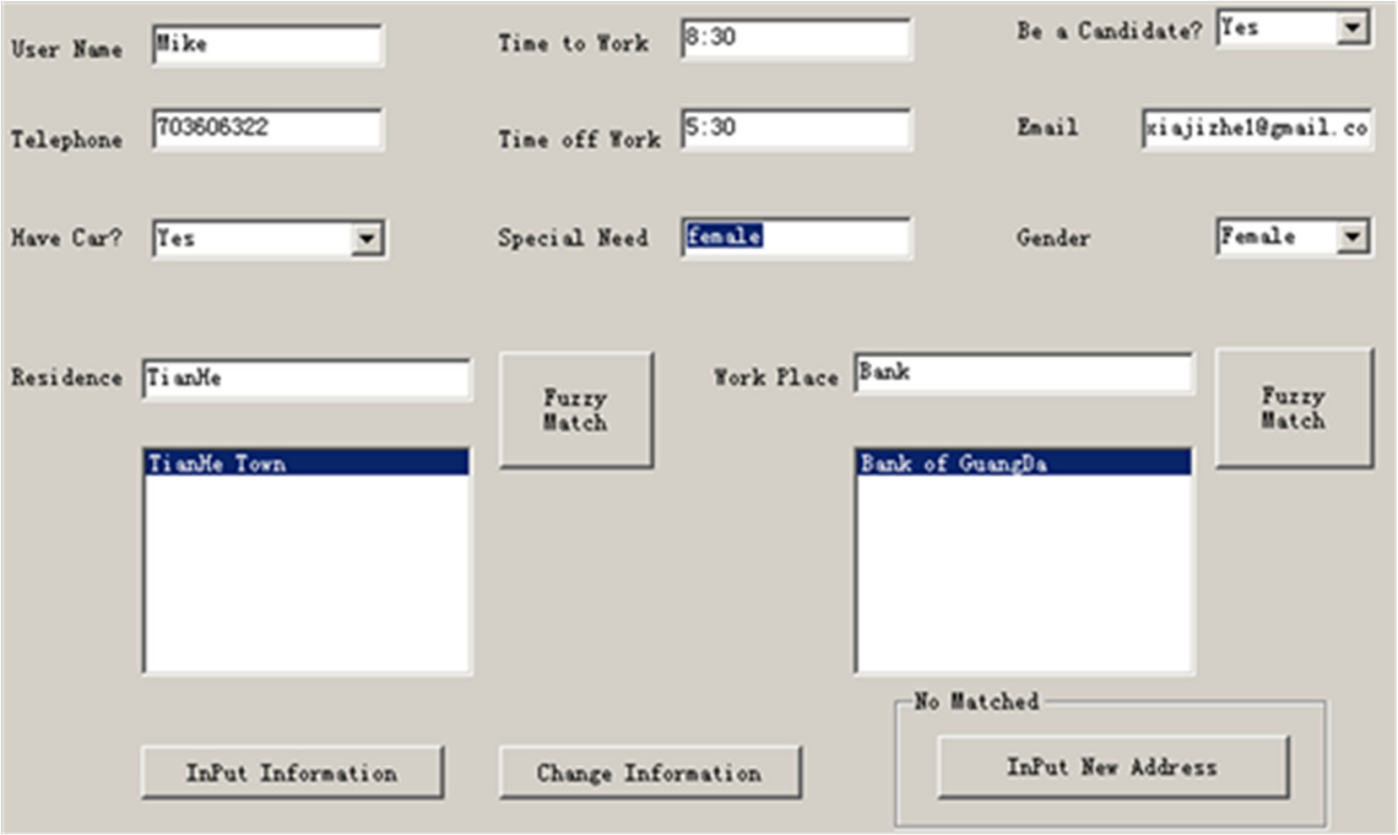
Registration Interface Design (allow this image to be published CC BY 3.0 license).

**Fig 9:**
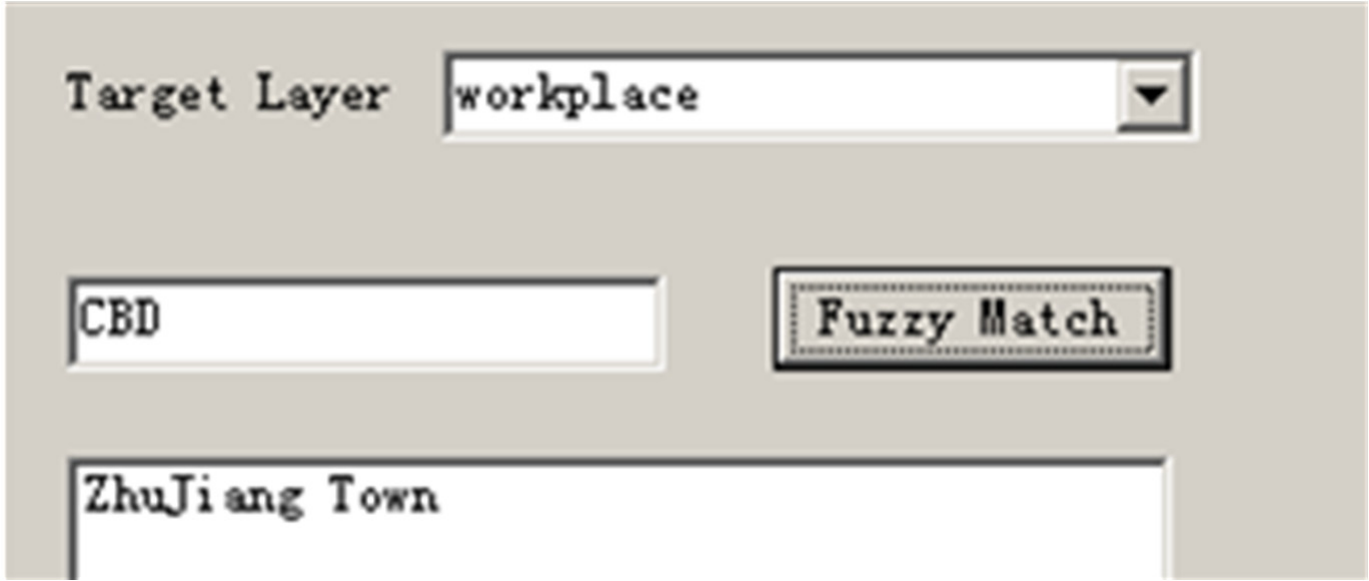
Alias match function (allow this image to be published CC BY 3.0 license).

Users must input required or optional information and register with the system. Registration input includes spatial, temporal, and text-based information (Fig 8). A user must input the location of their residence and their workplace (spatial data), their work schedule (temporal data), and text-based information such as user name, gender, age, and any special requirements (e.g. female only). Address geocoding is employed to transfer text-based residence and workplace inputs into spatial locations. In this example, an alias name database is used to better support the geocoding process. For example, when a user inputs the address “CBD”, meaning the downtown area, the system will automatically match to “ZhuJiang Town” (Fig 9).

#### Attribute Filter

In this process, the user filters candidates according to text-based and temporal requirements. For example, a user may only want to carpool with female candidates whose schedule is 9:00a.m.-5:30p.m.

#### Spatial Filter

**Fig 10:**
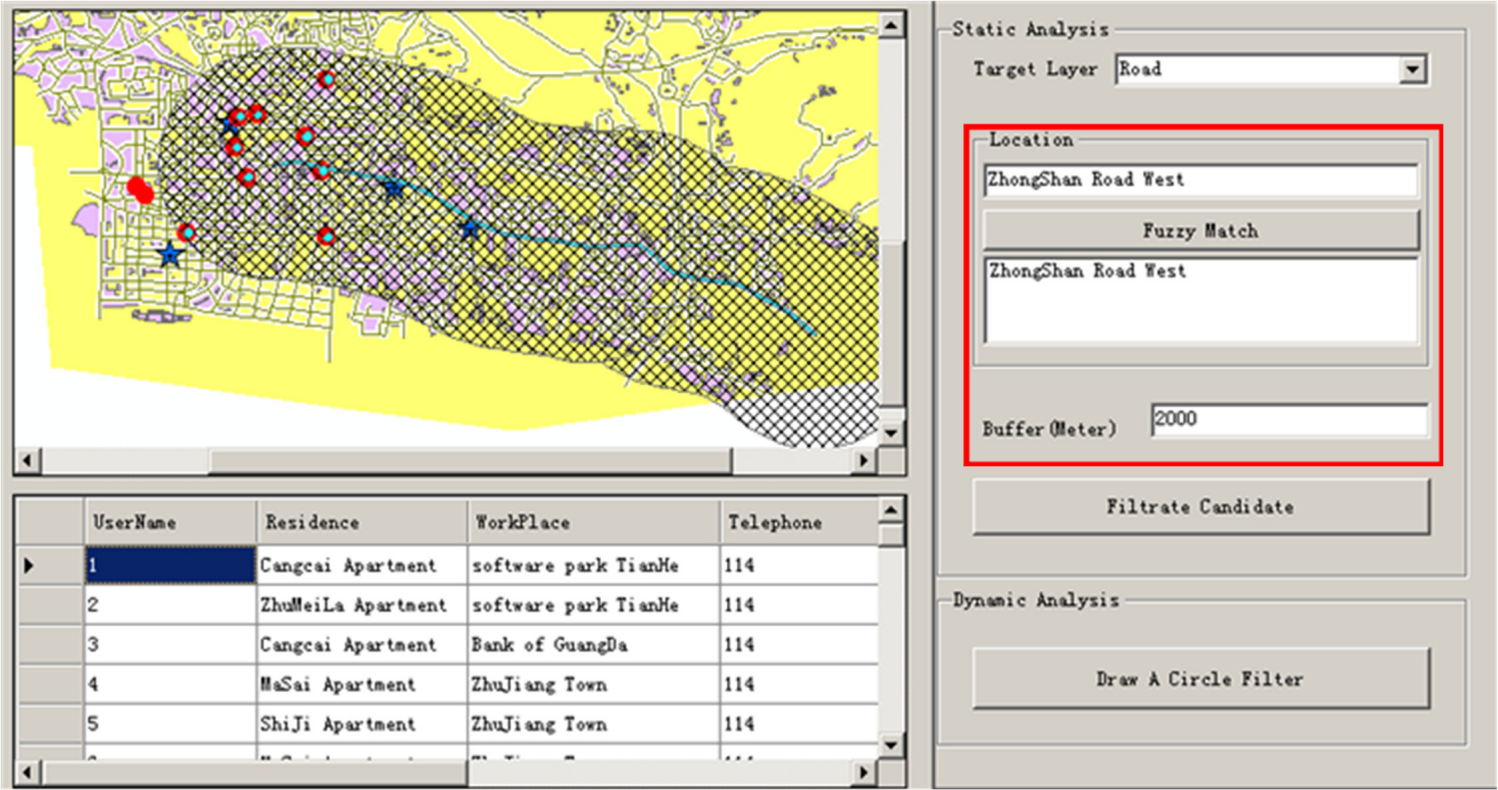
Spatial Filter (allow this image to be published CC BY 3.0 license).

In this process, the user may filter candidates according to spatial requirements. For example, a user may only want to carpool with candidates who live within 2000 meters of the Zhongshan Road West (Fig 10).

#### Determine carpooling passengers and daily commute route

**Fig 11:**
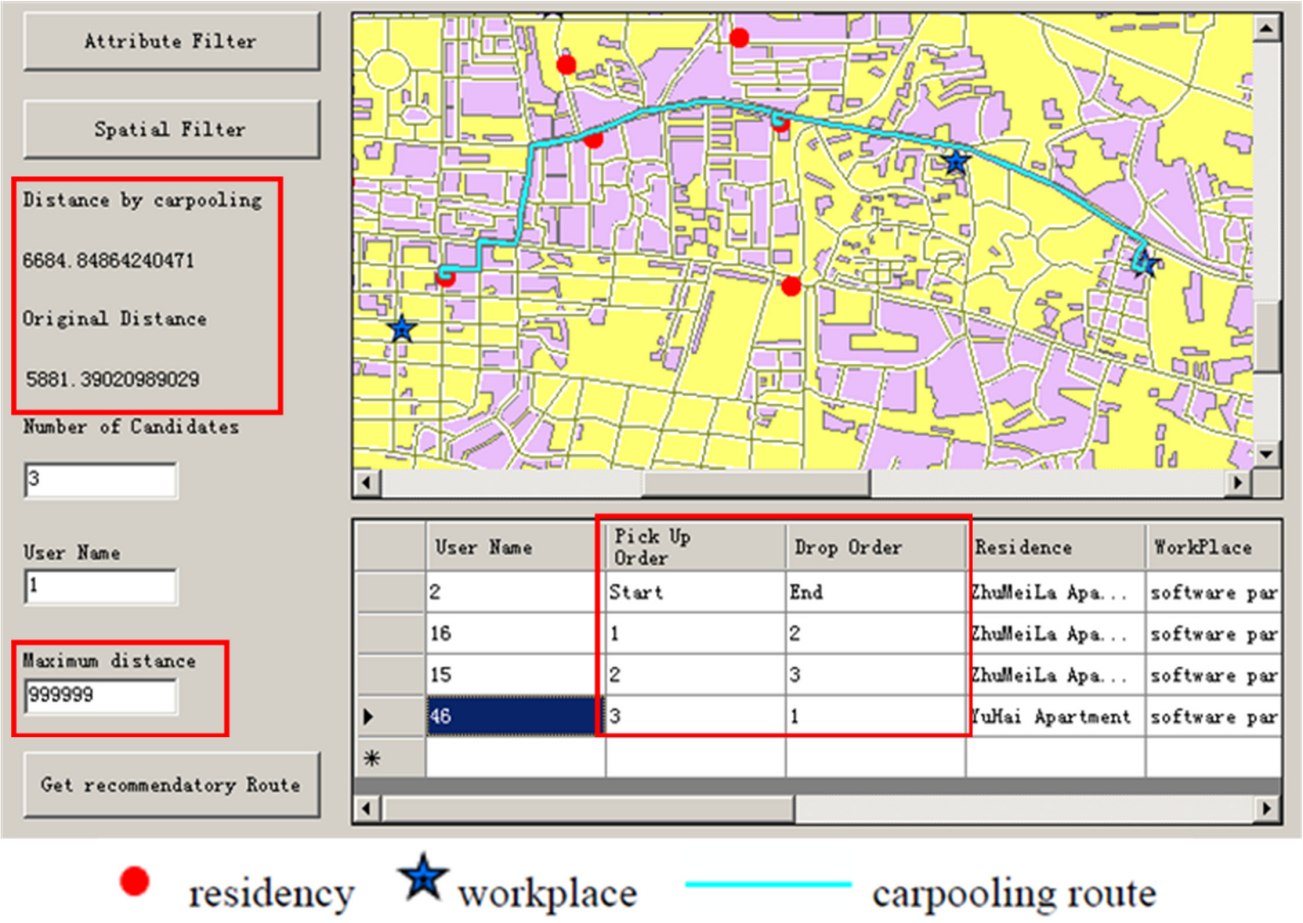
Carpool matching function (allow this image to be published CC BY 3.0 license).

After the attribute and spatial filters, the user must input the number of people in the carpooling team and the maximum commute distance. Then, the system executes the carpool matching model (based on the inputs above) and provides a recommended carpooling team. Information regarding recommended team members is shown in the system, such as user name, email, home address, workplace, etc. The daily carpooling commute route is shown on the map, specifying the order in which to pick up and drop off passengers. Moreover, the original commute distance (without carpooling), and the carpooling commute route distance are shown in the system so that the user can know the travel cost incurred through carpooling (Fig 11). If a passenger (e.g., P2) refuses the ride, the system will update the carpool route by choosing a back-up route that does not include passenger P2.

## Conclusion and future work

One purpose of this research was to understand the issues regarding the provision of carpool matching services. From the literature we know that carpooling is an important element of the transportation structure of large cities, and we know that there are matching and solution issues that must be addressed in any implementation. The research results do–in fact–expand our understanding in a manner that allows developers going forward to tune their carpool matching services to the kinds of problems that need to be solved (e.g. daily commute matching v. long term carpool determination). Specifically, this research provides an examination of a range of solution procedures that are appropriate under varying conditions. A carpool matching system prototype was constructed to demonstrate the efficacy of these methods. Perhaps most importantly, this research has shown that optimal 3+ person teams can be constructed with their associated routes for many common commute situations. This solution to a known technological problem represents a contribution to both the literature and practice of carpooling.

Several avenues for future research immediately present themselves. First, the finding that brute force search was nearly as effective as linear programming over a significant number of problem instances was surprising. This suggests that there is something idiosyncratic about the structure of this problem that lends itself to enumeration for a particular range of the parameters. If this can be generalized to other problems, that finding may change some approaches to practical problem-solving. Second, we know from the literature that ideas such as “comfort”, culture, and level of trust are important in the success of carpooling activities. It may be that elements of social network analysis, qualitative research, and even psychology could be incorporated into a more sophisticated carpool matching model, in order to increase the carpooling population, improve carpool efficiency, and construct more lasting carpool teams. A related issue is the incorporation of more complex cost measures, including individual passenger costs, which may vary based on the pick-up and drop-off order. A related issue is that of directional variation in cost. It is well known that the costs to traverse a given arc can be very different based on the direction of travel, particularly during periods of heavy traffic (e.g. rush hour). If variable costs based on direction were implemented through the addition of arcs to the network, that could significantly increase the complexity of any given problem instance. Other means of integrating variable costs (directional, temporal, or otherwise) could similarly create greater difficulty in finding optimal solutions. Since these issues are so common they deserve immediate attention in future research. Finally, while the testing of methods, and even the construction of the prototype, can be accomplished in the research computer lab, any implementation must instead be exposed to the true population of a metropolitan area via the internet. That test will bring with it significant challenges of a different nature from what was undertaken in this research. It is hoped that the results presented here will encourage researchers to pursue these and other problems regarding carpool matching.

## Supporting Information

S1 File DataAvailabilityStatement.doc. The Data Availability Statement
(DOCX)

S2 File ExperimentalData1.zip. The distance matrixes of randomly generated sets of potential passengers ranging from 50 to 700 passengers, incremented by 50
(ZIP)

S3 File ExperimentalData2.zip. The distance matrixes of randomly generated sets of potential passengers ranging from 750 to 900 passengers, incremented by 50
(ZIP)

S4 File ExperimentalData3.zip. The distance matrixes of randomly generated sets of potential passengers ranging from 900 to 1,000 passengers, incremented by 50, and the street network for Guangzhou city, China ESRI Geodatabase format
(ZIP)
