# Supplementary material for: A New Model for a Carpool Matching Service
Source: PLoS One. 2015 Jun 30;10(6):e0129257. doi: 10.1371/journal.pone.0129257 (PMC4488330; doi:10.1371/journal.pone.0129257)
Supplement: S1 File — The Data Availability Statement (DOCX) [file pone.0129257.s001.docx]

**Data Availability Statement**

**“A new model for a carpool matching service”**

**Supplemental Files**

The data sets necessary to reproduce the results presented in this article consists of the following:

1. The street network for Guangzhou city, China ESRI Geodatabase format
2. The distance matrixes of randomly generated sets of potential passengers ranging from 50 to 1,000 passengers, incremented by 50 (20 sets of randomly generated passenger location distances)

Due to the limited size of these datasets they are included with the submission as Supplemental Files.
